# Scat as a source of DNA for population monitoring

**DOI:** 10.1002/ece3.9415

**Published:** 2022-10-30

**Authors:** Jeffrey A. Manning, Taylor Edwards, John Clemons, Daniel J. Leavitt, Caren S. Goldberg, Melanie Culver

**Affiliations:** ^1^ School of the Environment Washington State University Pullman Washington USA; ^2^ University of Arizona Genetics Core, University of Arizona Tucson Arizona USA; ^3^ School of Natural Resources and the Environment, Conservation Genetics Laboratory University of Arizona Tucson Arizona USA; ^4^ U.S. Fish and Wildlife Service Arizona Ecological Services Field Office Phoenix Arizona USA; ^5^ U.S. Geological Survey, Arizona Cooperative Fish and Wildlife Research Unit University of Arizona Tucson Arizona USA

**Keywords:** fecal DNA, microsatellites, mitochondrial DNA, non‐invasive sampling, reptile, scat

## Abstract

Sampling fecal droppings (scat) to genetically identify individual animals is an established method for monitoring mammal populations and could be highly useful for monitoring reptile populations. Whereas existing protocols for obtaining DNA from reptile scat focus on analyses of whole, fresh scat deposited during animal handling, the collection of scat naturally deposited by reptiles in situ, as required for non‐invasive population monitoring, requires protocols to extract highly degraded DNA. Using surface swabs from such scats can reduce PCR inhibition and increase genotyping success. We report on three related but independently designed studies of DNA analyses from scat swabs of herbivorous reptiles under natural desert conditions: two free‐ranging desert tortoise species (Agassiz's desert tortoise, *Gopherus agassizii*, California, US, and Morafka's desert tortoise, *G. morafkai*, Arizona, US) and the common chuckwalla (*Sauromalus atar*) (Arizona, US, and Sonora, MX). We analyzed samples from both tortoise species with the same set of 16 microsatellites and chuckwalla samples with four mtDNA markers; studies also varied in swab preservation medium and DNA extraction method. Microsatellite amplification success per sample, defined as ≥9 loci with amplification, was 15% for the study of Agassiz's desert tortoise and for the study of 42% Morafka's desert tortoise. For chuckwallas, we successfully amplified and sequenced 50% of samples. We recovered fragments up to 400 bp for tortoises and 980 bp for chuckwallas from scat swab samples. This study indicates that genotypes can successfully be obtained from swabs of scat from herbivorous reptiles collected in the field under natural environmental conditions and emphasizes that repeat amplifications are necessary for the genetic identification of individuals from non‐invasive samples.

## Graphical abstract

Non‐invasive methods for monitoring herbivorous reptile populations would benefit our knowledge of their ecology, space use, and conservation status. Current methods of obtaining fecal DNA for these species have only been demonstrated to work well on fresh scats rather than those found under field conditions. We present a compilation of three independently conducted but similar studies of fecal DNA collected from the common chuckwalla, Agassiz's desert tortoise, and Morafka's desert tortoise to show that swabs of scats obtained under natural conditions have the potential to be used to obtain genotypes for population monitoring.

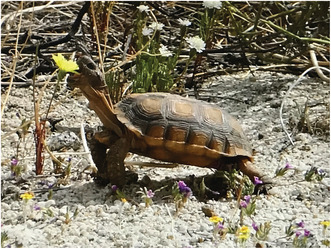

## 1 INTRODUCTION

Conservation of biological diversity relies on ingenuity and novel solutions to complex problems (Berger‐Tal & Lahoz‐Monfort, 2018). Understanding the dynamics of wildlife populations is critical for conservation but challenging for species that are difficult to observe and where individuals have few distinguishing markings. Non‐invasive methodological approaches to detect the presence and estimate population parameters for at‐risk species have been developed to fill this gap for many mammals (e.g., Ochoa et al., 2016; Ramón‐Laca et al., 2015; Woodruff et al., 2015), with a wide array of population‐level applications (Schwartz et al., 2006; Taberlet & Luikart, 1999), but methods for reptiles are relatively underdeveloped. Reptile populations are declining globally, and many are threatened with extinction (Gibbons et al., 2000; Stanford et al., 2020). Development of non‐invasive techniques for population monitoring within this taxonomic group would be an important contribution to their conservation and management.

Reptile scat sampling for DNA acquisition has generally relied on immediate, semi‐invasive collection of freshly deposited scat during direct disturbance or handling of animals (Jones et al., 2008; Mitelberg et al., 2019; Pearson et al., 2015; Yuan et al., 2015), while non‐invasive monitoring techniques require sampling of scats under field conditions. Desert environments, which often support high reptile species diversity, may provide some protection against DNA degradation by virtue of dryness, but this may be overcome by exposure to heat and UV, which degrade DNA. For example, amplification success for individually identifying microsatellites in Sonoran pronghorn (*Antilocapra americana sonoriensis*) scat dropped from 81 to 2% between days 1 and 14 under full‐sun desert field conditions (Woodruff et al., 2015). For desert tortoises, Mitelberg et al. (2019) found that fresh scat samples worked well (52% of samples amplified at all 6 screening loci) but that three samples experimentally exposed to sun for 7–9 days amplified for 17–67% of loci and did not meet their microsatellite genotyping quality standards with the methods tried.

To identify the species or individual that deposited a scat, DNA is typically obtained from the scat's outer coating, which contains epithelial cells shed from the intestinal lining (Stenglein et al., 2010; Wehausen et al., 2004). To date, all published methods for extracting DNA from reptile scat include the collection and extraction of the whole scat, while methods for collection from mammal scat include those focused on swabbing the outside surface of the scat (Rutledge et al., 2009). For herbivorous reptiles, the material from inside the scat may contain PCR inhibitors, as found in Pearson et al. (2015), which are likely to be released during the extraction process as the scats break up. The variety of DNA in the matrix can also lead to non‐specific amplification, even for herbivorous species (Marasinghe et al., 2021). These issues may be reduced by using surface swabs. The benefits of field‐swabbing scats versus collecting whole scat also include reduced weight and volume to carry from the field and reduced risk of zoonotic pathogen transport and exposure. Additionally, scats may play an important ecological or social role (Bull et al., 1999) in the environment, especially in desert environments with limited resources (e.g., Sanchez et al., 2004). The disadvantages of not collecting whole scat include inability to examine diet, viral/disease load, hormones, or other features at a later time.

We investigated the efficacy of genotyping DNA isolated from the outer coating of scat naturally deposited by reptiles in situ that had undergone environmental exposure for up to several months prior to collection, representing the scat samples with varying degrees of DNA degradation that would be available for long‐term population monitoring. We used scats that were opportunistically obtained during three separate studies of herbivorous reptiles in North America's Sonoran and Colorado Desert regions: Morafka's desert tortoise (*Gopherus morafkai*), Agassiz's desert tortoise (*G. agassizii*), and common chuckwalla (*Sauromalus ater*). While these studies were conducted independently, we present them here in combination to better contribute to the development of methods for collecting genotype data from field samples of herbivorous reptile scats under realistic conditions. We set out to answer three questions: (1) is it possible to obtain amplifiable DNA from swabs of herbivorous reptile scat exposed to field conditions? (2) is amplification from DNA samples derived from these swabs biased toward smaller fragments? and (3) how does allelic diversity compare between scat swabs and blood samples?

## 2 MATERIALS AND METHODS

### 2.1 Sample collection

**FIGURE 1:**
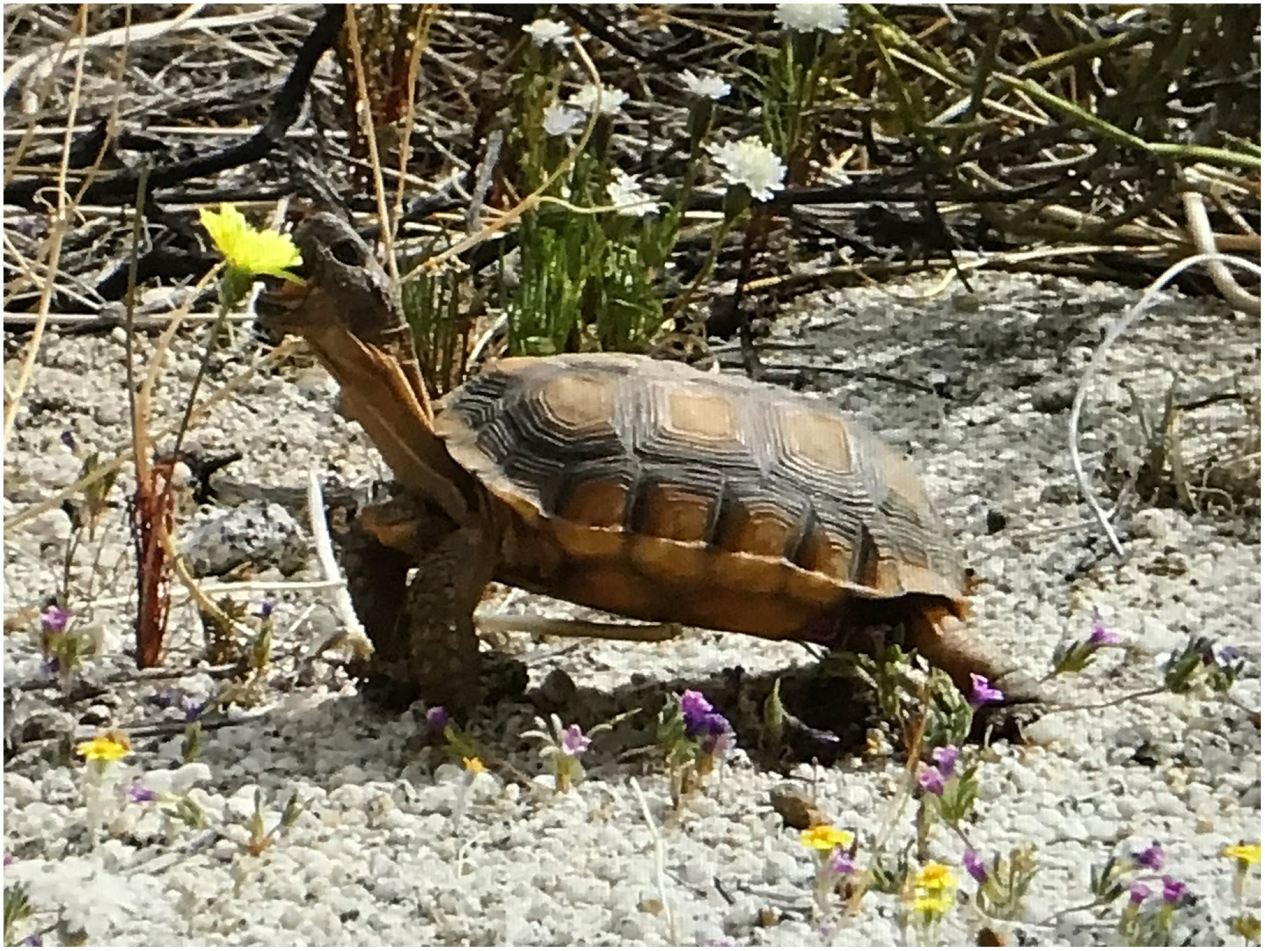
Agassiz's desert tortoise, *Gopherus agassizii*, in Anza‐Borrego Desert State Park. Photo by Jeff Manning.

We obtained scat samples from ongoing population studies of Morafka's desert tortoise in Florence Military Reservation (FMR), Arizona, USA (*N* = 38) between April and November 2012 and scat swab samples from Agassiz's desert tortoise in Anza‐Borrego Desert State Park (ABDSP; Figure 1), California, USA (*N* = 48) in the spring and summer of 2018. We also obtained blood samples from Agassiz's desert tortoise from the ABDSP during spring 2018 (*N* = 36). Common chuckwalla scat swabs were collected from two locations: Organ Pipe Cactus National Monument (ORPI), Arizona, USA (*n* = 4), and near Bahía la Cholla (BLC), Sonora, Mexico (*n* = 10). Chuckwalla scat swabs were collected from ORPI in May 2018 and were potentially from the scats deposited in the previous season of chuckwalla activity (Fall 2017); samples had visible bleaching on the top side.

Researchers in each study area collected scats opportunistically from under rock piles, in burrows, and in open exposed areas. Scats in this study contained a bulk of plant material and a glossy, outer coating. While we targeted sampling of fresher scats based on “shiny” appearance, some of the field samples were potentially weeks or months old. Each site contained scats from a broad range of environmental exposure constituting samples of variable quality indicative of real samples available for long‐term population monitoring. We stored Agassiz's desert tortoise and chuckwalla scats with silica bead packets individually in plastic baggies or 50 ml tubes and brought them to a field station (or laboratory) for swabbing. Morafka's desert tortoise scats were collected in paper coin envelopes and brought to the laboratory, placed in Ziploc bags, and frozen at −20°C.

### 2.2 DNA extraction

Each of the three studies used a slightly modified variant of Rutledge et al.’s (2009) scat swabbing protocol to obtain and isolate DNA from epithelial cells. This process consisted of moistening a cotton swab with a lysis or saline buffer and rubbing or rolling across the surface area of the dry scat to rehydrate the scat surface and gently transfer epithelial cells to the swab. For the Agassiz's desert tortoise and common chuckwalla samples, we rubbed the scat surface with a buccal swab (Whatman OmniSwab, WB100035; Whatman Inc., Clifton, NJ, USA) dampened with lysis buffer (50 mM Tris pH 8.0; 50 mM EDTA; 25 mM Sucrose; 100 mM NaCl; 1% SDS). This lysis buffer has been demonstrated to be highly effective for preserving and recovering buccal DNA from these swabs (Goldberg et al., 2003). For Morafka's desert tortoise, the frozen scats were swabbed with a cotton‐tipped applicator dampened with 1× solution of phosphate‐buffered saline (PBS; 1.37 M NaCl, 0.027 M KCl, 0.119 M phosphate buffer) rolled over several locations of the scat surface, then placed directly in 300 μl ATL buffer and 66 μl proteinase K (Qiagen Inc., Valencia, CA, USA) and incubated at 21°C overnight, followed the next day by extraction. This swabbing method has been demonstrated to have high success at recovering target species' DNA from scat (Vynne et al., 2012).

For Agassiz's desert tortoise swabs and blood and common chuckwalla swabs, we isolated total DNA by overnight lysis with proteinase K at 55°C, followed by robotic extraction using a QIAGEN BioSprint 96 robotic magnetic‐particle purification system (Qiagen) and Aline Biosciences Buccal Swab gDNA Kit protocol (Aline Biosciences, Woburn, MA, USA). We quantified recovered DNA for Agassiz's desert tortoise and common chuckwalla with a BioTEK Synergy HT (BioTEK, Winooski, VT, USA). This automated protocol was not available for Morafka's desert tortoise swabs because these extractions were performed in a separate study; we extracted these samples following the QIAmp DNeasy Tissue Kit protocol (Qiagen Inc.), which uses spin columns rather than magnetic beads to capture DNA. All extractions were conducted within 6 months of collection. Extractions for Morafka's desert tortoise included extraction negative controls.

### 2.3 Evaluation of amplifiable DNA

Microsatellite genotyping was performed at the University of Arizona Genetics Core in Tucson, Arizona USA. We tested Morafka's desert tortoise samples for amplification at 16 microsatellite loci (Edwards et al., 2003; Schwartz et al., 2003), following protocols described in Murphy et al. (2007). For Agassiz's desert tortoises, a 7‐locus screening set (subset from the 16 described above) was used on all samples; only samples that produced genotypes for at least three of these seven loci were moved forward in genotyping the additional nine loci used in Morafka's desert tortoise plus nine more microsatellite loci (as in Edwards & Berry, 2013). For common chuckwalla, microsatellites were unavailable, and we instead targeted the cytochrome b region of the mitochondrial genome. We used two published primer pairs which amplify 855 and 980 bp of DNA, respectively (Corl et al., 2010; Kocher et al., 1989). In addition, because we suspected samples had experienced DNA degradation, we designed primer pairs from mitochondrial sequences downloaded from the GenBank database (GenBank Accession no. AF020234; Petren & Case, 1997) that amplified 338 and 352 bp, respectively: SAAT_*Cytb*_338, fwd‐GGCCTCTACTACGGCTCCTA and rev‐GAGTTGAGTCCGGTTGGGTT, SAAT_*Cytb*_352 fwd‐AGCC TTCTCATCAGTTGCCC and rev‐GAATCGAGTTAGGGTCGCGT. Sample results were evaluated using agarose gels and Sanger sequencing. DNA extractions and PCR preparation were conducted in a space separate from where PCR products are handled, and a negative control was included with each plate of reactions for all species. All PCRs were conducted once for each sample at a locus (not using a multi‐tube approach) due to logistical constraints.

### 2.4 Tests for size bias in amplification

We tested for evidence of null alleles and scoring error due to stuttering with program Micro‐Checker (version 2.2.3; Van Oosterhout et al., 2004) for all desert tortoise samples. For chuckwallas, we evaluated the success (defined as the recovery of expected sequence) of each targeted size fragment.

### 2.5 Allele counts from blood and scat samples

Scat sample collection for both desert tortoise species was paired with analyses of blood samples from the study population (Agassiz's desert tortoise, *N* = 36) or a nearby population (Morafka's desert tortoise; *N* = 8). Blood samples of Agassiz's desert tortoise were concurrent with scat sampling, while blood samples collected from Morafka's desert tortoise were from approximately a decade prior (Edwards et al., 2004). We compared number of alleles generated for each locus between the sample types to investigate the data loss from these samples.

## 3 RESULTS

### 3.1 Evaluation of amplifiable DNA

**FIGURE 2:**
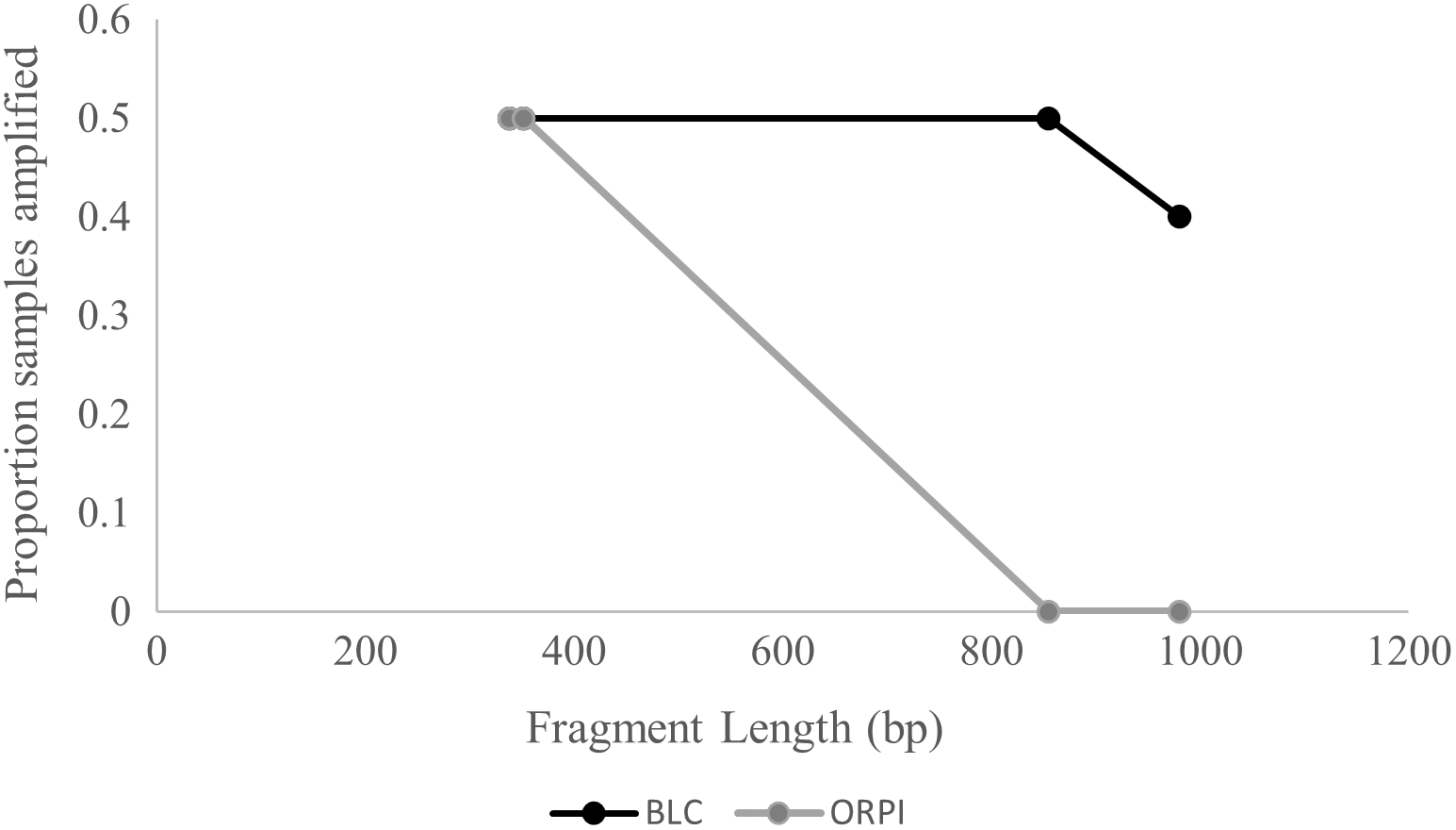
Amplification success of DNA isolated from swabs of scat samples from common chuckwalla (*Sauromalus ater*) for different length loci of the cytochrome b (cytb) region of the mitochondrial genome. Samples were collected from near Bahía la cholla (BLC), Sonora, Mexico, and at organ pipe cactus National Monument (ORPI), Arizona, US.

Of the 38 Morafka's desert tortoise scat samples, 21 amplified some genotype data. Per locus amplification rates ranged from 18.42–57.89% (Table 1), and 17 of the samples produced amplification at ≥9 STRs (42% of samples). Nine of the 48 Agassiz's desert tortoise scat samples from ABDSP passed the initial screening and were genotyped at 25 loci; of those seven produced amplicons for ≥9 STRs (15% of all samples) with per locus amplification success of 0–50.00% (Table 1). For chuckwallas, we generated successful PCR amplicons for seven of 14 (50%) of the extracted samples (Figure 2). Agassiz's desert tortoise samples quantified at between 0.15–14.71 ng/μl (mean = 3.99 ng/μl; total yield 33.75–3310 ng) and samples that amplified successfully ranged from 2.35–14.32 ng/μl. For chuckwallas, total DNA was quantified at 0–4.89 ng/μl (mean = 2.05 ng/μl, total yield 0–1222.5 ng) and samples that amplified successfully ranged from 0.35 to 4.89 ng/μl. All negative controls tested negative and no additional amplicons were observed that would indicate sample contamination.

**TABLE 1:** Proportion of amplifying microsatellites relative to expected size range of amplicon for Morafka's desert tortoise (blood *N* = 8, scat *N* = 38) and Agassiz's desert tortoise (blood *N* = 36, scat *N* = 48, scat after initial screening *N* = 9).

Locus	Size (bp)	Morafka's desert tortoise			Agassiz's desert tortoise		
		Blood	Scat	ΔAllele count	Blood	Scat	ΔAllele count
		% Amplified	% Amplified		% Amplified	% Amplified	
Goag4	128–195	100	36.84	4	67.57	12.50*	3
Cm58	130–134	100	23.68	0	97.30	0*	1
GP96	143–151	100	47.37	0	97.40	55.56	0
Goag32	176–186	100	57.89	‐2	91.89	50.00*	0
GP61	190–256	100	52.63	2	97.30	66.67	3
GP30	194–228	100	50.00	2	94.59	66.67	−1
GP15	198–282	100	44.74	7	94.59	44.44	9
GP19	232–260	100	50.00	0	94.59	55.56	0
GP26	253–259	100	23.68	1	86.49	0	2
Goag5	257–364	100	50.00	−1	97.30	45.83*	1
Goag7	258–282	100	39.47	0	94.59	33.33*	2
GP55	264–314	100	36.84	1	97.30	55.56	2
GP102	302–371	100	52.63	1	97.30	11.11	7
Goag6	356–440	100	18.42	6	81.08	10.42*	15
Goag3	369–385	100	50.00	1	94.59	25.00*	0
GP81	370–402	100	42.11	−1	89.19	11.11	4
	Median	100%	46.06%	1	94.59%	25.00%	2

*Note*: The median value for % loci amplified from scat samples for Agassiz's desert tortoise was calculated only for the seven loci where amplification was attempted for all samples, indicated by *. ΔAllele count is the count of alleles amplified from blood samples minus the count of alleles amplified from scat samples. For Agassizi's desert tortoise this was calculated from only samples that passed the first screening.

### 3.2 Tests for size bias in amplification

For Morafka's desert tortoise, seven loci showed possible signs of a null allele and no evidence of scoring error due to stutter. The sample size was too low for this evaluation in Agassiz's desert tortoise genotypes. For the mtDNA amplifications of chuckwalla samples, five samples from BLC amplified for the 855 bp locus and four amplified for the 980 bp locus, while the maximum amplicon length for ORPI was two samples that generated a product for the 352 bp locus (Figure 2).

### 3.3 Allele count from blood and scat samples

Data generated from these single‐well amplifications of scat samples recovered generally lower numbers of alleles than blood samples from the same or close‐by populations, with a greater difference for Agassiz's desert tortoise (Table 1).

## 4 DISCUSSION

This study demonstrates that useful amounts of DNA can be successfully isolated from swabs of herbivorous reptile scat collected in the field under natural field conditions. Despite the expectation that DNA extracted from scat should exhibit greater degradation and fragmentation than DNA from fresh tissue, our results did not reveal patterns of amplification failure of larger microsatellite amplicons up to 400 bp, although beyond that we did observe better amplification of smaller amplicons in at least one population of chuckwallas.

Our amplification success rate was lower for Agassiz's desert tortoises (15%), where swabs were collected from dried samples and preserved in lysis buffer, than for Morafka's desert tortoises (42%), where scats were stored frozen and swabs were collected in PBS immediately prior to DNA extraction. Morafka's desert tortoises were analyzed at fewer loci (16 vs. 25), so this is a minimum estimate of these differences. These numbers bracket those achieved using DNA extractions of desert tortoise whole scat (31% for field‐exposed samples; Mitelberg et al., 2019). Similar success (31%) was found for swabs of otter scat in an initial year of study, which was doubled in subsequent years by increasing the area of scat swabbed and swabbing the area twice with a rinse in the lysis buffer storage tube in between (Klütsch & Thomas, 2018). This latter method approaches the success that Miles et al. (2015) found for canid whole scat extraction (74%), which was more than double that from microsatellite genotyping of fecal swabs preserved in ethanol from the same study (35%; Miles et al., 2015). Canid DNA (estimated using quantitative PCR) was also found to be more concentrated in whole scat extractions than in scat swab samples (average 0.85 ng/μl vs. 0.06 ng/μl; Miles et al., 2015). We found comparatively higher yields in this study (average 2–4 ng/μl), but these were quantified from whole extracts and so included an unknown quantity of bacteria and other non‐target organisms. We recovered long fragments of mitochondrial DNA from chuckwalla scats using the same methods as for Agassiz's desert tortoise. The greater success of the chuckwalla samples may have been due to the greater abundance of mitochondrial versus nuclear DNA and/or the smaller size of chuckwalla scats leading to more effective preservation using silica beads.

Sampling using swabs of scats may improve the cost‐efficiency of extraction methods and avoid inhibitors contained inside scats. Swabs are more easily extracted using automated methods, as was done in this study for Agassiz's desert tortoise. Automated methods were found to be as successful and more cost‐effective than processing whole scats for genetic detection of the invasive Australian red fox (Quasim et al., 2018). Inhibitors can be an important issue for amplification success; Pearson et al. (2015) found that plant material inside lizard scats may interfere with PCR and Miles et al. (2015) found that extractions from whole canid scats were more inhibited than swab scat samples. Microsatellite genotype success was 100% in a study using whole scats from herbivorous Arabian oryx (Ochoa et al., 2016) but these samples originated from a zoo and were collected when fresh.

Differences in success between tortoise species in this study could be due to age of samples, swabbing technique, preservation method, and/or different extraction methods. There was also an overall lower success rate for Agassiz's desert tortoise demonstrated by the blood samples that could be related to bias in the loci used, but that does not account for most of the differences between the results for the two tortoise species. Comparative studies are needed to optimize this technique for field application, but this work demonstrates that swabbing of reptile scats has the potential to produce useful information for non‐invasive population monitoring.

This preliminary work was performed to test techniques that could be applied to samples collected under realistic field conditions. We found amplifiable DNA in scat swabs for the full range of microsatellite sizes (128–402 bp) and up to 980 bp for mtDNA. Mitochondrial DNA may be more robust to degradation than nuclear DNA, and these results may indicate that long reads of mtDNA may be available from field‐collected scat samples. Because of funding and logistical constraints, samples were only analyzed once for each locus, rather than the multi‐tube approach that is standard for microsatellite analyses of non‐invasive samples (Taberlet et al., 1999). Therefore, even samples that appear to be successfully genotyped may have allelic dropout or false alleles, as is commonly found in genotyping of scat samples (Broquet et al., 2007) and is consistent with our generally lower allele counts in scat versus blood samples from the same or close by populations. However, this work adds to our knowledge of methods for efficiently collecting and extracting DNA from scat samples of herbivorous reptiles (and likely other species), demonstrating that swab samples from scats collected under realistic field conditions can be used to establish genetic monitoring programs that apply further quality control to achieve consensus genotypes.

## AUTHOR CONTRIBUTIONS

**Jeffrey A. Manning:** Conceptualization (equal); funding acquisition (equal); investigation (equal); methodology (equal); writing ‐ original draft (equal); writing ‐ review and editing (equal). **Taylor Edwards:** Conceptualization (equal); formal analysis (lead); investigation (equal); methodology (equal); writing – original draft (equal). **John Clemons:** Formal analysis (supporting); investigation (equal); writing – original draft (supporting). **Daniel J. Leavitt:** Conceptualization (equal); funding acquisition (equal); investigation (equal); methodology (equal); writing – original draft (supporting). **Caren Goldberg:** Formal analysis (supporting); writing – original draft (equal); writing – review and editing (equal). **Melanie Culver:** Conceptualization (equal); funding acquisition (equal); investigation (equal); resources (supporting); writing – original draft (supporting).

## CONFLICT OF INTEREST

The authors declare no conflicts of interest.

## Data Availability

DNA sequences (common chuckwalla): Genbank accessions ON601071, ON601072. Microsatellite results (desert tortoises): Dryad https://doi.org/10.5061/dryad.qrfj6q5js.
