# Corrigendum

**DOI:** 10.1002/ece3.9629

**Published:** 2023-01-16

In the recent article by Manning et al. (2022), the authors would like to correct the article title, so it reads as follows:

Scat as a source of DNA for monitoring herbivorous reptile populations.
